# Evaluating the Impact of Climate Change on the Asia Habitat Suitability of *Troides helena* Using the MaxEnt Model

**DOI:** 10.3390/insects16010079

**Published:** 2025-01-14

**Authors:** Fengrong Yang, Quanwei Liu, Junyi Yang, Biyu Liu, Xinqi Deng, Tingjiang Gan, Xue Liao, Xiushan Li, Danping Xu, Zhihang Zhuo

**Affiliations:** 1 College of Life Science, China West Normal University, Nanchong 637002, China; youngyanglil@foxmail.com (F.Y.); quanwei66977@foxmail.com (Q.L.); yang.junyi@foxmail.com (J.Y.); biyuliuql@foxmail.com (B.L.); deng.xinqi@foxmail.com (X.D.); sy_0914@foxmail.com (X.L.); xiushanli@vip.163.com (X.L.); danpingxu@foxmail.com (D.X.); 2 Engineering Research Centre of Chuanxibei Rural Human Settlement (RHS) Construction, Mianyang Teachers’ College, Mianyang 621016, China; gantinjiangky@mtc.edu.cn

**Keywords:** *Troides helena*, MaxEnt, climate change, potential suitable distribution, species conservation

## Abstract

Butterflies are highly sensitive to climate change, and *Troides helena*, as an endangered butterfly species, is also affected by these changes. To enhance the conservation of *T. helena* and effectively plan its protected areas, it is crucial to understand the potential impacts of climate change on its distribution. This study utilized a MaxEnt model in combination with ArcGIS technology to predict the global potential suitable habitats of *T. helena* under current and future climate conditions, using the species’ distribution data and relevant environmental variables. The results indicated that the MaxEnt model provided a good prediction accuracy for the distribution of *T. helena*. Under the current climate scenario, the species is primarily distributed in tropical regions, with high suitability areas concentrated in tropical rainforest climates. In future climate scenarios, the suitable habitat areas for *T. helena* in medium and high suitability categories generally show an expansion trend, which increases over time. Especially under the SSP5-8.5 scenario, by the 2090s, the area of high suitability for *T. helena* is projected to increase by 42.85%. The analysis of key environmental factors revealed that precipitation of the wettest quarter (Bio16) was the most significant environmental factor affecting the distribution of *T. helena*. The species has high demands for precipitation and temperature and can adapt to future climate warming. This study is valuable for identifying the optimal conservation areas for *T. helena* and provides a reference for future conservation efforts.

## Simple Summary

*Troides helena*, a species within the Papilionidae family and Troides genus, was an endangered tropical butterfly in urgent need of conservation. The MaxEnt model was used to predict its global distribution, aiming to assess suitable future conservation measures. The results indicated that *T. helena* was primarily distributed in tropical regions, and its distribution range was projected to expand in the future. This study evaluated the distribution of *T. helena*, provided conservation recommendations, and offered guidance for future conservation efforts.

## 1. Introduction

Global climate warming has become a proven fact, which has had a significant impact on the survival of biological communities [1,2] and has also altered the distribution range of species [3]. These impacts are generally negative, leading to a sharp decline in species distribution [4]. Studies have shown that the impact of climate change on the distribution of insects is significantly higher than that on other species [5,6]. Insects account for 76% to 98% of the global animal biomass [7], making them one of the most diverse and widely distributed animals in the biosphere, and they play an important role in ecosystem functions and services [8]. Like most species, insect biodiversity has decreased under climate influence [9], and their distribution range has been compressed. However, in certain regions, insect biomass has increased with the accumulation of heat [10]. As a type of insect, butterflies, due to their high sensitivity to environmental changes, are considered ecological indicator species [11], often used to monitor ecosystem health. The biodiversity of butterflies is also influenced by various environmental factors, and it has been confirmed that climate factors, particularly temperature and precipitation, are the main influences [12,13]. Although climate change has some harmful effects on tropical butterflies, they can adapt to temperature changes by regulating themselves [14]; therefore, tropical butterflies may exhibit different responses to environmental changes. *Troides helena*, belonging to the genus *Troides* in the family Papilionidae, is primarily distributed in tropical regions, with its larvae feeding on plants in the Aristolochiaceae family [15]. The adult butterfly plays a role in pollination, contributing to the genetic diversity of plants [16]. *T. helena* has strict habitat requirements, particularly being sensitive to microclimatic conditions and vegetation cover. Its population and distribution changes often reflect fluctuations in environmental quality [17,18]. It is listed as a nationally protected species of Level II in the “National List of Key Protected Wild Animals”. In the face of the threats posed by climate change, the conservation of endangered butterfly species, such as *T. helena*, is essential. Studying the impact of climate change on the distribution range of *T. helena* contributes to its conservation efforts, which not only helps maintain species diversity but also benefits the health of habitat ecosystems.

In the conservation of species’ habitats, evaluating the suitable habitat areas based on environmental variables is of great significance [19]. Currently, species distribution models (SDMs) are widely used in ecology and conservation biology to study species distribution [20]. These models predict potential distribution areas by analyzing the relationship between species’ geographic distribution data and environmental variables [21]. SDMs include various methods, with the MaxEnt model being the most widely used [22]. It can be applied in fields such as biodiversity, invasive species management, species conservation, and protected area planning and management [23]. The MaxEnt model was first proposed in 2004 [24], and it is based on the maximum entropy principle. It estimates the ecological requirements of a species and simulates its potential distribution using species presence data and environmental variables [25]. Due to its advantages, such as low data requirements, high prediction accuracy, and ease of use, it has stood out among many species distribution models and has been widely applied [26,27]. Previously, the MaxEnt model has been extensively used in the conservation of Lepidoptera species. For example, Hernández-Baz et al. used the model to predict the potential geographic distribution of the Mexican endemic moth *Coreura albicosta* [28]. Pilar Fernández et al. utilized the MaxEnt model to predict that the overwintering habitats of the monarch butterfly in coastal California might shift to higher altitudes due to climate change [29]. Such studies not only provide scientific evidence for species conservation but also demonstrate the advantages of the MaxEnt model in addressing complex environmental changes. Today, the MaxEnt model has become an indispensable tool in the fields of ecology and conservation biology. Using the MaxEnt model to study species distributions helps understand and protect biodiversity, holding significant scientific and practical value.

Current research on *T. helena* mainly focuses on material characteristics [30,31], with limited studies on its distribution [15]. Furthermore, there is a need for further research on the adaptability of *T. helena*’s habitat, which has restricted the conservation efforts for this species to some extent. This study employed the MaxEnt model and ArcGIS v 10.8 software to predict the potential geographical distribution of *T. helena* globally, assess the impact of various environmental parameters on its distribution, explore the suitability of current and future habitats, and analyze the relationship between environmental variables and the species, in order to identify key conservation areas for *T. helena*. The aim was to provide a scientific theoretical foundation for future conservation efforts for *T. helena*.

## 2. Materials and Methods

### 2.1. Species Distribution Data

The establishment of the MaxEnt model requires that accurate and sufficiently abundant distribution point data be available. The distribution point data used in this study were obtained from the following three sources: (1) the Global Biodiversity Information Facility (GBIF Occurrence Download https://doi.org/10.15468/dl.xw4wnb, accessed on 8 August 2024); (2) the National Animal Specimen Resource Center (http://museum.ioz.ac.cn/, accessed on 8 August 2024); and (3) by searching relevant literature using “*Troides helena*” as a search term in CNKI (https://www.cnki.net/, 8 August 2024) and Web of Science (https://www.webofscience.com/wos/, accessed on 8 August 2024), from which distribution records of *T. helena* were obtained. The coordinates were then obtained through Google Maps (https://maps.google.com/, accessed on 8 August 2024). After obtaining the coordinates, to reduce the impact of data redundancy between the coordinate points, spatial filtering was applied to the coordinates using ENMTools v 1.4. Only one data point was retained in each minimum grid (4.5 × 4.5 km) [32]. A total of 514 distribution point data for the species were obtained.

### 2.2. Selection of Environmental Variables

A total of 22 environmental variables were selected in this study (Table S1). Among them, 19 bioclimatic variables were obtained from the WorldClim 2.1 dataset (http://www.worldclim.org/, accessed on 8 August 2024), which was constructed based on the Coupled Model Intercomparison Project 6 (CMIP6) of the IPCC’s Sixth Assessment Report, and 3 topographic variables were obtained from the National Centers for Environmental Information (NCEI, https://www.ngdc.noaa.gov/, accessed on 8 August 2024). All variables were selected based on the version released in 2023. This study selected three scenarios for modeling: the sustainable development scenario (SSP1-2.6), the moderate scenario that maintains development with no changes (SSP2-4.5), and the high greenhouse gas emission scenario with no control measures (SSP5-8.5) [33]. Not all environmental variables were used in the model construction, as an excessive number of variables could lead to issues such as multicollinearity, overfitting, and reduced interpretability. A Pearson correlation analysis was performed on the 22 selected environmental variables (Table S2), and variables with correlation coefficients (*r*) < |0.7| were selected [34]. Ultimately, 7 environmental variables were retained (Table 1).

### 2.3. Model Optimization

To improve the accuracy of model predictions, we adjusted two parameters: the regularization multiplier (RM) and feature combination (FC) [35,36]. First, the distribution point data and related environmental variables for *T. helena* were selected. Then, 25% of the data were randomly assigned as a test set, with the remaining 75% used as the training set. The RM and FC parameters were adjusted using the R package kuenm. Five feature combinations (FC) and corresponding regularization multipliers (RM) were set, generating five candidate models. These models were tested using the kuenm package, and the best model was selected based on the following two criteria: omission rate (OR) < 5% and Delta AICc (Akaike Information Criterion) < 2. Finally, the model with the smallest Delta AICc value was selected as the optimized model, which corresponds to the best parameter settings for the regularization multiplier and feature combination.

### 2.4. Model Evaluation and Habitat Suitability Classification

In the study, the accuracy of the MaxEnt model simulation results was typically evaluated using AUC (Area Under the ROC Curve). The AUC value ranges from 0.5 to 1, with larger values indicating better model performance [37]. The modeling results were visualized using ArcGIS v10.8 software, simulating the geographical distribution map of *T. helena*’s suitable habitat under current and future climate conditions. The suitable habitat was classified into four categories: unsuitable areas (0–0.173), low suitability areas (0.173–0.347), moderate suitability areas (0.347–0.521), and high suitability areas (0.521–1), based on the maximum training sensitivity threshold [38,39]. Subsequently, ArcGIS tools were used to analyze the changes in the high-suitability areas and centroids of *T. helena* under current and future conditions.

## 3. Results

### 3.1. Modeling Results and Key Environmental Factors Affecting T. helena

**Figure 1:**
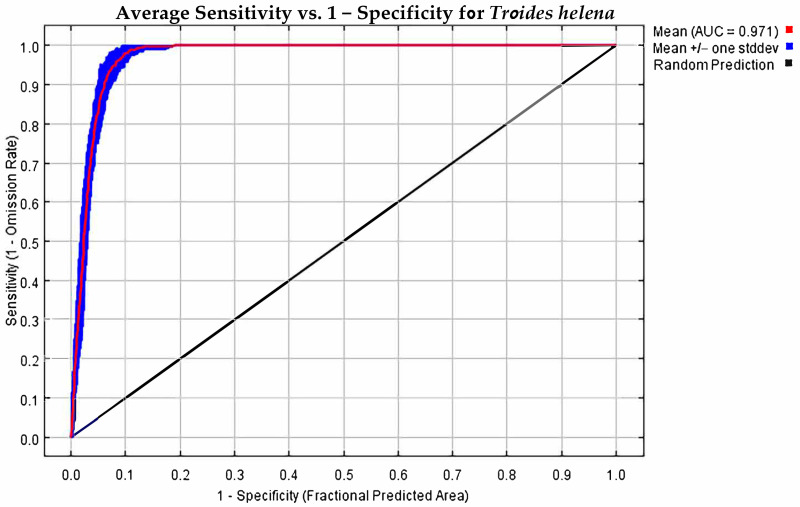
Evaluation of the modeling results for *T. helena* using the ROC curve and AUC.

**Figure 2:**
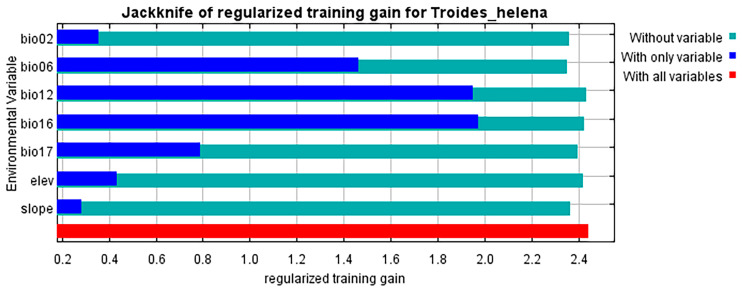
Variable importance, as determined via the folding jackknife test, for *T. helena*.

After 10 repeated runs, the final model used to predict *T. helena* had an AUC of 0.971 for both AUC_training_ and AUC_test_ (Figure 1, Table S3) and the model omission rate was 0.021, indicating that the MaxEnt model has high predictive performance for the distribution of *T. helena* and can be used to construct a suitability distribution model for the species on a global scale. Based on the analysis of the importance and contribution rate of each environmental variable, the five most significant factors affecting the distribution of *T. helena*, ranked by contribution rate, are as follows (Table S4): Precipitation of Wettest Quarter (Bio16, 55.1%), Annual Precipitation (Bio12, 25.4%), Min Temperature of Coldest Month (Bio6, 4.7%), and the average diurnal temperature range (Bio2, 4.5%), which together account for 89.7% of the total contribution. Based on the score from the jackknife method (Figure 2, Table S4), the top four most important environmental factors are Min Temperature of Coldest Month (Bio6, 52.5%), Precipitation of Wettest Quarter (Bio16, 19.6%), average diurnal temperature range (Bio2, 14.8%), and Precipitation of Driest Quarter (Bio17, 6.5%). Finally, we chose to discuss Precipitation of Wettest Quarter, Annual Precipitation, Min Temperature of Coldest Month, and the average diurnal temperature range.

### 3.2. Analysis of the Potential Suitable Habitat of T. helena Under Current Climate Conditions

**Figure 3:**
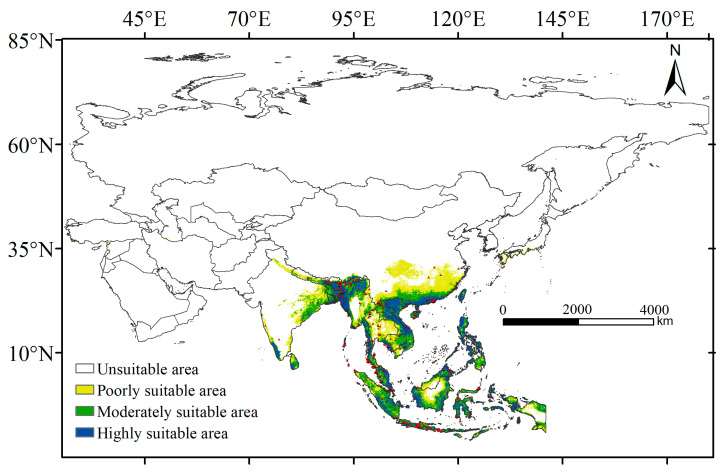
Potential distribution and occurrence records of *T. helena* under current climate conditions. The red triangles represent the occurrence records of *T. helena*.

According to Figure 3, the modeling results show a range slightly larger than the actual range of *T. helena* occurrence. The high-suitability habitat areas for *T. helena* are primarily located in the tropics, with a wide distribution across East Asia, Southeast Asia, and South Asia (Figure 3), covering a total area of 1514.13 × 10^3^ km^2^ (Table 2). The potential suitable habitat (Figure 3) closely matches the current distribution records of the species (Figure 3), mainly occurring in China, Myanmar, Cambodia, Thailand, Vietnam, the Philippines, Malaysia, Indonesia, as well as in Eastern India and Bangladesh in South Asia. The medium-suitability areas are predominantly found in the tropical and subtropical regions, covering an area of 1851.44 × 10^3^ km^2^ (Table 2), with a broad distribution in China within Asia. The low-suitability areas, which are similar to the medium-suitability areas but are located further from the tropics, are mainly found in subtropical regions, covering a total area of 2102.08 × 10^3^ km^2^ (Table 2). Overall, *T. helena* has a wide distribution in tropical regions and is suited to environments with high temperatures and abundant precipitation.

### 3.3. Prediction of Suitable Habitats for T. helena Under Future Climate Conditions

**Figure 4:**
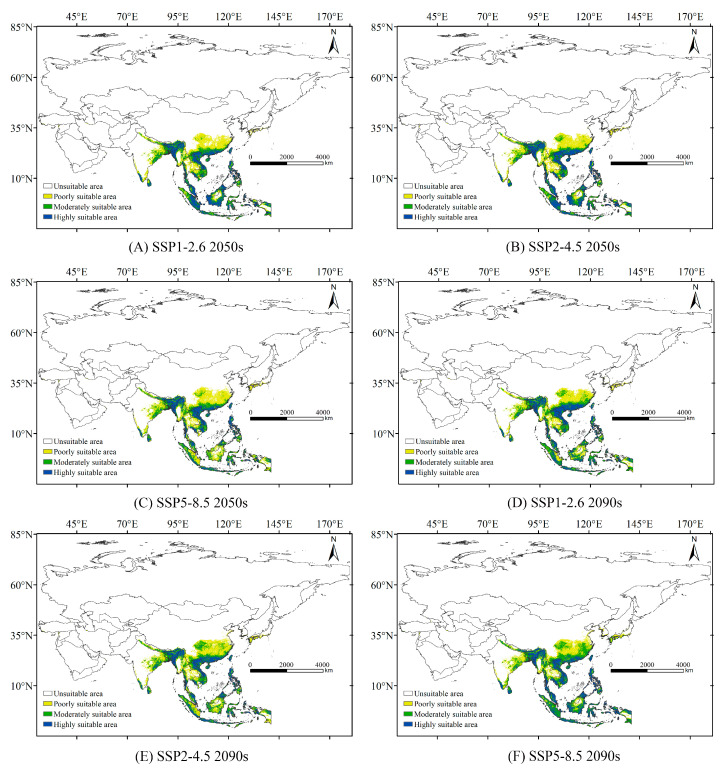
Potential distribution of *T. helena* in future periods (2050s, 2090s) under the SSP1-2.6, SSP2-4.5, and SSP5-8.5 climate change scenarios. (**A**) represents the potential suitable habitat under the SSP1-2.6 scenario in the 2050s, (**B**) represents the potential suitable habitat under the SSP2-4.5 scenario in the 2050s, (**C**) represents the potential suitable habitat under the SSP5-8.5 scenario in the 2050s, (**D**) represents the potential suitable habitat under the SSP1-2.6 scenario in the 2090s, (**E**) represents the potential suitable habitat under the SSP2-4.5 scenario in the 2090s, and (**F**) represents the potential suitable habitat under the SSP5-8.5 scenario in the 2090s.

As shown in Figure 4, the potential distribution of *T. helena* under different future climate scenarios was simulated. The analysis revealed that the suitable habitat of *T. helena* varied under different scenarios. In the 2050s, the area of high suitability habitat changed between 4.01% and 38.28% (Table 2), with larger variations observed in southern China and Southeast Asia (Figure 4A–C). The area of moderate suitability habitat decreased, with the reduction ranging from 3.66% to 7.57% (Table 2). Additionally, the area of low suitability habitat increased, with the increase ranging from 0.09% to 26.94% (Table 2). Notably, under the SSP5-8.5 scenario in the 2050s (Table 2), the area of moderate suitability habitat increased the most, while the reduction in high suitability habitat was the greatest. By the 2090s, the area of high suitability habitat decreased under the SSP1-2.6 and SSP2-4.5 scenarios compared to the 2050s, but under the SSP5-8.5 scenario, the area increased the most compared to the present, reaching 42.85% (Table 2). The area of moderate suitability habitat also increased, with the largest increase observed under the SSP5-8.5 scenario, reaching 23.28% (Table 2). Overall, the suitable habitat of *T. helena* is expected to expand in the future, with high suitability habitats remaining primarily in tropical regions, while some suitable habitats may expand towards higher latitudes.

### 3.4. Changes in the High Suitable Habitats of T. helena from Present to Future

**Figure 5:**
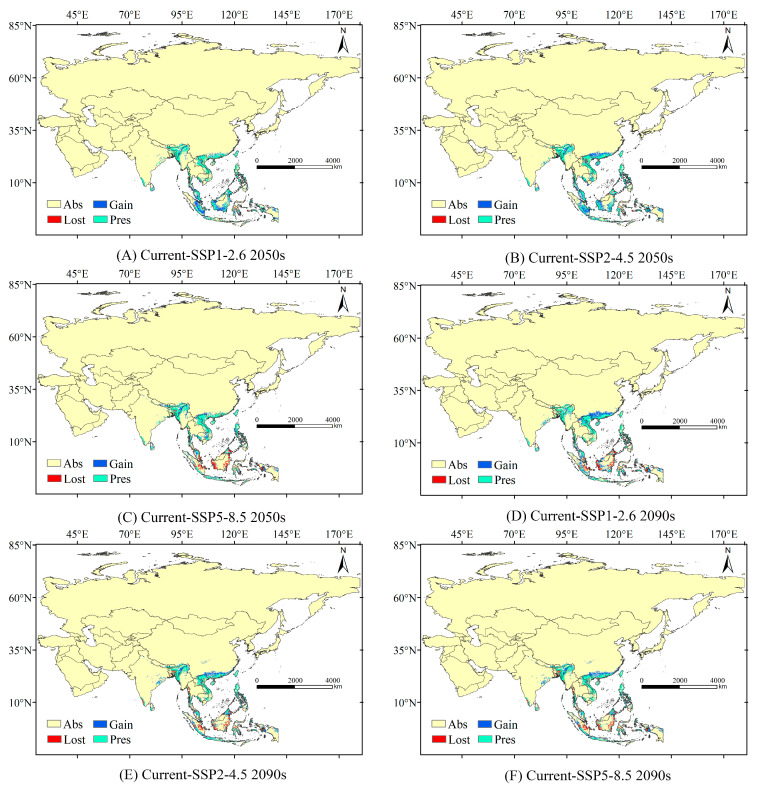
Changes in the High Suitability Habitat of *T. helena* from the Present to the Future; “Gain” indicates areas where high suitability habitats have increased, “Lost” indicates areas where high suitability habitats have decreased, “Abs” indicates the area where non-high suitability habitats (unsuitable, low, and medium suitability) remain unchanged, and “Pres” indicates the area where high suitability habitats remain unchanged. (**A**) represents the changes in potential suitable habitat from the current scenario to the SSP1-2.6 scenario in the 2050s, (**B**) represents the changes in potential suitable habitat from the current scenario to the SSP2-4.5 scenario in the 2050s, (**C**) represents the changes in potential suitable habitat from the current scenario to the SSP5-8.5 scenario in the 2050s, (**D**) represents the changes in potential suitable habitat from the current scenario to the SSP1-2.6 scenario in the 2090s, (**E**) represents the changes in potential suitable habitat from the current scenario to the SSP2-4.5 scenario in the 2090s, and (**F**) represents the changes in potential suitable habitat from the current scenario to the SSP5-8.5 scenario in the 2090s.

The contraction and expansion of the high suitability habitat for *T. helena* in the future is shown in Figure 5. In the 2050s, the changes in high suitability habitat for *T. helena* were mainly observed in Southeast Asia, particularly in Indonesia and Malaysia, with some expansion in India (Figure 5A–C). The area of expansion reached its maximum under the SSP2-4.5 scenario, with an increase of 670.56 × 10^3^ km^2^ (Table 3). Additionally, the area of contraction was smallest under the SSP2-4.5 scenario, with an increase of only 90.97 × 10^3^ km^2^ (Table 3). In the 2090s, the high suitability habitat for *T. helena* showed signs of migrating to higher altitudes, with a notable expansion within China. The expansion reached its maximum under the SSP5-8.5 scenario, with an increase of 840.02 × 10^3^ km^2^ (Table 3). Furthermore, significant contraction was observed in Indonesia and Malaysia (Figure 5D–F), with the smallest contraction area under the SSP1-2.6 scenario, with an increase of only 647.73 × 10^3^ km^2^ (Table 3).

### 3.5. Response Analysis of Dominant Environmental Variables

**Figure 6:**
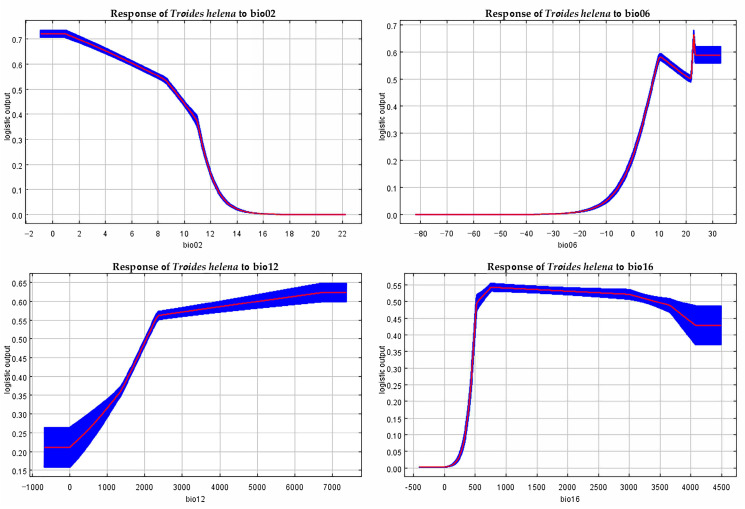
Response curves of *T. helena* to the five dominant environmental variables. The blue area represents the range of occurrence probability, while the red curve represents the average occurrence probability.

Based on the response curves of environmental variables to the distribution probability of *T. helena* in the MaxEnt model (Figure 6 and Figure S1), the suitable environmental variable ranges for the potential distribution of *T. helena* were determined (Table 4 and Table S5). The suitable ranges and optimal values for the environmental variables for *T. helena* are as follows: mean diurnal range was −0.84 to 6.17 °C (Bio2, optimal value: 1.02 °C), minimum temperature of the coldest month was 22.25 to 23.29 °C (Bio6, optimal value: 22.88 °C), annual precipitation was 2156.04 to 7355.73 mm (Bio12, optimal value: 6690.76 mm), and precipitation of the wettest quarter was 636.18 to 2965.72 mm (Bio16, optimal value: 758.00 mm). The distribution probability of *T. helena* increased with the increasing values of environmental variables but began to decrease once the optimal value was reached. Notably, except for the mean diurnal range, *T. helena* maintained a high distribution probability regardless of how the other three environmental variables changed.

## 4. Discussion

### 4.1. Evaluation and Prediction Results of the MaxEnt Model

In this study, the MaxEnt model was used to predict the potential suitable habitat areas for *T. helena* worldwide, based on its distribution data and 10 environmental variables, for both current and future periods. The accuracy of the model was evaluated using the AUC, and the average AUC value of the MaxEnt model was 0.971, indicating that the model provided good predictions of the distribution area of *T. helena*, with high reliability [25]. The model predictions show that the current potential suitable habitat for *T. helena* is located in the tropical rainforests and coastal areas of East Asia, where high temperatures and abundant precipitation provide a favorable environment. The total area of the high-suitability habitat is 1514.13 × 10^3^ km^2^. Under different future scenarios, the high-suitability habitat for *T. helena* is projected to increase over time, consistent with findings from studies on other tropical insects [40,41]. The analysis of current and future changes suggests a trend of northward migration of the species’ suitable habitat, a pattern that has been confirmed by several previous studies [42,43].

### 4.2. Key Environmental Variables and Ecological Characteristics

The study, by analyzing the key environmental factors affecting the occurrence probability of *T. helena*, derived the species’ response curves to these environmental variables. The results indicate that precipitation is a key factor influencing the distribution of *T. helena*, including precipitation of wettest quarter (Bio16, 55.1%) and annual precipitation (Bio12, 25.4%), which together contribute a total of 80.5% to the species’ distribution. Additionally, the min temperature of coldest month (Bio6, 4.7%) and mean diurnal temperature range (Bio2, 4.5%) also play significant roles in shaping the distribution of *T. helena*.

Some studies have shown that in areas with latitudes lower than 40°, precipitation is more likely to become the dominant factor limiting butterfly survival [44], a result consistent with our study. Precipitation contributes more to the distribution than temperature and is the primary environmental factor influencing its distribution. A reduction in precipitation may lead to decreased larval hatching rates and habitat degradation [45,46]. This study indicates that *T. helena* requires higher levels of precipitation, as even with precipitation levels above its suitable range, the occurrence probability remains relatively high, suggesting a significant demand for precipitation. Research has shown that insect abundance is positively correlated with precipitation [47]. Therefore, drought, as an important factor limiting insect diversity [48], may also be a crucial condition limiting the distribution of *T. helena*. Temperature is also an important environmental variable affecting the distribution of *T. helena*. The min temperature of coldest (Bio6) and the mean diurnal range (Bio2) were found to have a significant impact on the distribution of *T. helena*. Body temperature was a key factor influencing butterfly survival, and butterflies needed to maintain their body temperature within a suitable range [49]. As small ectothermic animals, butterflies were highly dependent on the environment for thermoregulation and were, therefore, vulnerable to temperature fluctuations [50]. In the tropics, where temperatures were consistently high, the suitable temperature range for *T. helena* was also relatively high, with the min temperature of coldest ranging from 8.29 to 18.52 and 22.19 to 23.29 °C, which was much higher than the suitable range for non-tropical species like the monarch butterfly [51], explaining why *T. helena* was primarily distributed in tropical regions. Research has shown that butterflies in warmer regions adapted to higher temperatures, which could lead to population increases in the future under climate warming [52], consistent with the results of this study showing an expansion of high suitability areas for *T. helena* in tropical regions.

Under the current scenario, the distribution area of *T. helena* was concentrated in the tropics. In the future, the suitable areas for *T. helena* would expand, especially in tropical rainforest climates and coastal areas, further validating that precipitation and temperature were the major factors affecting butterflies, with environments of high temperature and high precipitation being suitable for their survival. This study contributed to understanding the relationship between environmental factors and *T. helena* populations, determining its distribution range, and promoting the conservation of butterfly species diversity.

### 4.3. Limitations of the Model and Species Conservation

The MaxEnt model is a species distribution prediction tool based on the maximum entropy principle, widely applied in ecological research and species conservation planning [53]. Studies have shown that *T. helena* is widely distributed in tropical rainforest climates and may experience range expansion in the future, which is beneficial for its conservation. However, MaxEnt modeling cannot account for all factors [54]. For instance, it does not consider the distribution of host plant species, and changes in climate, such as warming and variations in precipitation, can affect plant–herbivore interactions, thereby influencing the distribution of animals [55]. Like other species in the butterfly family, *T. helena* is oligophagous, primarily feeding on Aristolochiaceae plants [15], and its distribution range is largely constrained by the availability of host plants [56]. Therefore, even if the climate is suitable, *T. helena* may not be able to thrive in certain areas if sufficient food sources are lacking. The inability to account for such factors limits the model’s accuracy, often leading to predicted suitable habitats that exceed the actual distribution range [57]. Thus, when applying modeling results, it is essential to consider both species characteristics and local environmental conditions comprehensively. The projected increase in the suitable habitat for *T. helena* does not imply that its population will be free from risk; conservation efforts are still needed, and people must join forces to contribute to its protection. The predicted increase in suitable habitats for *T. helena* in the future does not imply that the species will be free from danger. Conservation efforts still require significant contributions from people to protect the species. The high-suitability habitat of *T. helena* is relatively stable in tropical regions, while the success rate of ex situ conservation measures is lower [58]. Therefore, in situ conservation should be the primary focus for protecting *T*. *helena*. Establishing nature reserves is the optimal in situ conservation method. The selection of nature reserves should consider areas with stable future suitability, such as countries in China, Vietnam, eastern India, and Indonesia. Additionally, on-site investigations are necessary to ensure the presence of suitable host plant distributions in these areas [59]. This is because the plants consumed by the larvae are crucial for the distribution of *T*. *helena* [15]. Future conservation efforts should include the cultivation of Aristolochiaceae plants, increasing the availability of food sources for larvae and, thereby, improving the survival chances of *T*. *helena* in these areas [55,60]. Ongoing monitoring of the population distribution dynamics, in combination with key environmental factors, is essential for developing effective conservation strategies for *T*. *helena*.

## 5. Conclusions

This study successfully predicted the current and future potential suitable habitats of *Troides helena* using the MaxEnt model. The results indicate that future climate change could have a positive impact on the distribution of *T. helena*. Precipitation variables (Bio12 and Bio16) and temperature variables (Bio6 and Bio2) all influence the species’ distribution, with Bio16 contributing 55.1%, making it the most significant environmental factor. Under current conditions, the high-suitability habitat of *T. helena* is primarily distributed across China and Southeast Asia. In the future, the potential suitable habitat is expected to increase, especially under the SSP5-8.5 scenario, where the area of high-suitability habitat in the 2090s is predicted to increase by 42.85%. A comparison of current and future distribution changes reveals that the increase in high-suitability habitat is most pronounced in China, while significant contractions occur in Indonesia and Malaysia. Based on the current distribution of *T. helena*, in situ conservation efforts should focus on countries such as China, Vietnam, eastern India, and Indonesia. This study evaluates the distribution patterns of *T. helena* and its future changes, providing valuable support for future conservation efforts.

## Figures and Tables

**Table 1:** Environmental variables affecting *T. helena* and host plants distribution.

Code	Variable	Unit
Bio2	Mean Diurnal Range	°C
Bio6	Min Temperature of Coldest Month	°C
Bio12	Annual Precipitation	mm
Bio16	Precipitation of Wettest Quarter	mm
Bio17	Precipitation of Driest Quarter	mm
elev	elev	m
Slope	slope	°

**Table 2:** Area of suitable habitats for *T. helena* under current and future climate conditions.

		Predicted Area (×10^3^ km^2^)			Comparison with Current Distribution (%)		
		Low Habitat Suitability	Medium Habitat Suitability	Highly Habitat Suitability	Medium Habitat Suitability	Low Habitat Suitability	Highly Habitat Suitability
Current		2102.08	1851.44	1514.13	\	\	\
2050s	SSP1-2.6	2103.91	1783.63	1938.35	0.09%	−3.66%	28.02%
	SSP2-4.5	2223.87	1711.35	2093.72	5.79%	−7.57%	38.28%
	SSP5-8.5	2668.35	1760.83	1574.81	26.94%	−4.89%	4.01%
2090s	SSP1-2.6	2269.25	1989.53	1769.24	7.95%	7.46%	16.85%
	SSP2-4.5	2604.64	2035.38	1655.59	23.91%	9.94%	9.34%
	SSP5-8.5	2111.65	2282.43	2162.88	0.46%	23.28%	42.85%

**Table 3:** Area of Changes in *T. helena* from Present to Future.

		Gain (×10^3^ km^2^)	Abs (×10^3^ km^2^)	Pres (×10^3^ km^2^)	Lost (×10^3^ km^2^)
2050s	SSP1-2.6	567.97	57,825.73	1370.38	143.75
	SSP2-4.5	670.56	57,723.14	1423.16	90.97
	SSP5-8.5	445.31	57,948.39	1129.50	384.64
2090s	SSP1-2.6	598.13	57,795.57	1171.11	343.02
	SSP2-4.5	532.99	57,860.71	1122.60	391.53
	SSP5-8.5	840.02	57,553.68	1322.86	191.27

**Table 4:** Suitable environmental variable ranges for the potential distribution of *T. helena*.

Environmental Variables	Suitable Range	Suitable Range
Bio2	−0.84–8.83 °C	1.02 °C
Bio6	8.29–18.52; 22.19–23.29 °C	22.88 °C
Bio12	2156.04–7355.73 mm	6690.76 mm
Bio16	636.18–2965.72 mm	758.00 mm

## Data Availability

The data supporting the results are available in a public repository at: https://figshare.com/s/974ad58c1e2b5a6d2ab6, 20 November 2024; GBIF.org (8 August 2024) GBIF Occurrence Download https://doi.org/10.15468/dl.xw4wnb, accessed on 8 August 2024.

## Supplementary Materials

The following supporting information can be downloaded at: https://www.mdpi.com/article/10.3390/insects16010079/s1, Figure S1: Response curves of *T. helena* to the five dominant environmental variables. Table S1. Lists the 19 bioclimatic variables used in the modeling process. Table S2. The Pearson correlation coefficient between environmental factors and *T. helena.* Table S3. AUC_training_ and AUC_test_ in modeling process. Table S4. Percentage contribution and ranking of environment variables in Maxent model. Table S5: Suitable environmental variable ranges for the potential distribution of *T. helena*.
