# Supplementary material for: Evaluating the Impact of Climate Change on the Asia Habitat Suitability of Troides helena Using the MaxEnt Model
Source: Insects. 2025 Jan 14;16(1):79. doi: 10.3390/insects16010079 (PMC11766371; doi:10.3390/insects16010079)
Supplement: Supplementary file 1 [file insects-16-00079-s001.zip › Attached table.pdf]

Table S1. Lists the 19 bioclimatic variables used in the modeling process.

Code	Environmental Variables	Unit
Bio1	Annual Mean Temperature	°C
Bio2	Mean Diurnal Range	°C
Bio3	Isothermality	%
Bio4	Temperature Seasonality	°C
Bio5	Max Temperature of Warmest Month	°C
Bio6	Min Temperature of Coldest Month	°C
Bio7	Temperature Annual Range	°C
Bio8	Mean Temperature of Wettest Quarter	°C
Bio9	Mean Temperature of Driest Quarter	°C
Bio10	Mean Temperature of Warmest Quarter	°C
Bio11	Mean Temperature of Coldest Quarter	°C
Bio12	Annual Precipitation	mm
Bio13	Precipitation of Wettest Month	mm
Bio14	Precipitation of Driest Month	mm
Bio15	Precipitation Seasonality	mm
Bio16	Precipitation of Wettest Quarter	mm
Bio17	Precipitation of Driest Quarter	mm
Bio18	Precipitation of Warmest Quarter	mm
Bio19	Precipitation of Coldest Quarter	mm
slope	slope	°
aspect	aspect	°
elev	elev	m

Table S2. The Pearson correlation coefficient between environmental factors and *Troides helena.*

	aspect	bio01	bio02	bio03	bio04	bio05	bio06	bio07	bio08	bio09	bio10	bio11	bio12	bio13	bio14	bio15	bio16	bio17	bio18	bio19	elev
slope	0.07	0.15	0.09	0.07	−0.05	0.15	0.14	−0.03	0.17	0.11	0.16	0.14	0.16	0.16	0.11	−0.12	0.16	0.12	0.21	0.09	0.08
aspect	0	0.1	0.05	0.03	−0.02	0.1	0.09	0	0.11	0.08	0.11	0.09	0.03	0.04	0.01	−0.06	0.04	0.01	0.04	0.02	−0.1
Bio01	0	0	0.59	0.7	−0.54	0.97	0.97	−0.36	0.95	0.92	0.98	0.98	0.54	0.54	0.31	−0.39	0.56	0.33	0.52	0.32	−0.7
Bio02	0	0	0	0.44	−0.16	0.66	0.49	0.12	0.56	0.53	0.61	0.54	0.02	0.1	−0.1	0.05	0.1	−0.11	0.1	−0.09	−0.17
Bio03	0	0	0	0	−0.85	0.55	0.8	−0.74	0.53	0.78	0.56	0.8	0.64	0.63	0.35	−0.05	0.64	0.38	0.48	0.48	−0.23
Bio04	0	0	0	0	0	−0.32	−0.71	0.96	−0.3	−0.73	−0.35	−0.7	−0.56	−0.56	−0.28	0.02	−0.56	−0.31	−0.36	−0.43	0.1
Bio05	0	0	0	0	0	0	0.89	−0.12	0.97	0.83	1	0.9	0.43	0.44	0.25	−0.41	0.46	0.26	0.46	0.22	−0.74
Bio06	0	0	0	0	0	0	0	−0.56	0.87	0.96	0.9	1	0.61	0.6	0.36	−0.35	0.62	0.38	0.53	0.39	−0.63
Bio07	0	0	0	0	0	0	0	0	−0.13	−0.58	−0.16	−0.54	−0.54	−0.51	−0.31	0.02	−0.51	−0.35	−0.32	−0.45	0.03
Bio08	0	0	0	0	0	0	0	0	0	0.76	0.98	0.88	0.47	0.47	0.29	−0.47	0.49	0.3	0.53	0.23	−0.77
Bio09	0	0	0	0	0	0	0	0	0	0	0.84	0.96	0.52	0.53	0.29	−0.29	0.54	0.31	0.4	0.37	−0.55
Bio10	0	0	0	0	0	0	0	0	0	0	0	0.92	0.46	0.46	0.28	−0.43	0.48	0.29	0.48	0.24	−0.76
Bio11	0	0	0	0	0	0	0	0	0	0	0	0	0.59	0.59	0.33	−0.33	0.61	0.35	0.52	0.37	−0.62
Bio12	0	0	0	0	0	0	0	0	0	0	0	0	0	0.91	0.75	−0.33	0.94	0.78	0.84	0.76	−0.37
Bio13	0	0	0	0	0	0	0	0	0	0	0	0	0	0	0.48	−0.11	0.99	0.51	0.78	0.62	−0.32
Bio14	0	0	0	0	0	0	0	0	0	0	0	0	0	0	0	−0.47	0.51	0.99	0.62	0.68	−0.28
Bio15	0	0	0	0	0	0	0	0	0	0	0	0	0	0	0	0	−0.16	−0.48	−0.3	−0.3	0.56
Bio16	0	0	0	0	0	0	0	0	0	0	0	0	0	0	0	0	0	0.55	0.81	0.64	−0.35
Bio17	0	0	0	0	0	0	0	0	0	0	0	0	0	0	0	0	0	0	0.64	0.71	−0.29
Bio18	0	0	0	0	0	0	0	0	0	0	0	0	0	0	0	0	0	0	0	0.42	−0.36
Bio19	0	0	0	0	0	0	0	0	0	0	0	0	0	0	0	0	0	0	0	0	−0.23

Table S3. $AUC_{training}$ and AUC_{test} in modeling process.

	Repeating Group										
	**1**	**2**	**3**	**4**	**5**	**6**	**7**	**8**	**9**	**10**	**Average**
$AUC_{training}$	0.973	0.973	0.973	0.973	0.972	0.973	0.973	0.974	0.973	0.973	0.971
AUC_{test}	0.970	0.976	0.974	0.967	0.979	0.980	0.968	0.960	0.969	0.965	0.971

Table S4. Percentage contribution and ranking of environment variables in Maxent model.

**Code**	**Variable**	**Percent Contribution**	**Permutation Importance**
bio16	Precipitation of Wettest Quarter	55.1	19.6
bio12	Annual Precipitation	25.4	2
bio06	Min Temperature of Coldest Month	4.7	52.5
bio02	Mean Diurnal Range	4.5	14.8
slope	slope	4.1	3.5
bio17	Precipitation of Driest Quarter	4.1	6.5
elev	elev	2.2	1.1

Table S5: Suitable environmental variable ranges for the potential distribution of *T. Helena*

Environmental variables	Suitable range	Suitable range
Bio17	13.35 – 225.31 mm	24.46 mm
elev	all	-669.50 - -105.47 m
slope	> 2.29 °	23.53 °
