# Supplementary figures and images for: Evaluating the Impact of Climate Change on the Asia Habitat Suitability of Troides helena Using the MaxEnt Model
Source: Insects. 2025 Jan 14;16(1):79. doi: 10.3390/insects16010079 (PMC11766371; doi:10.3390/insects16010079)

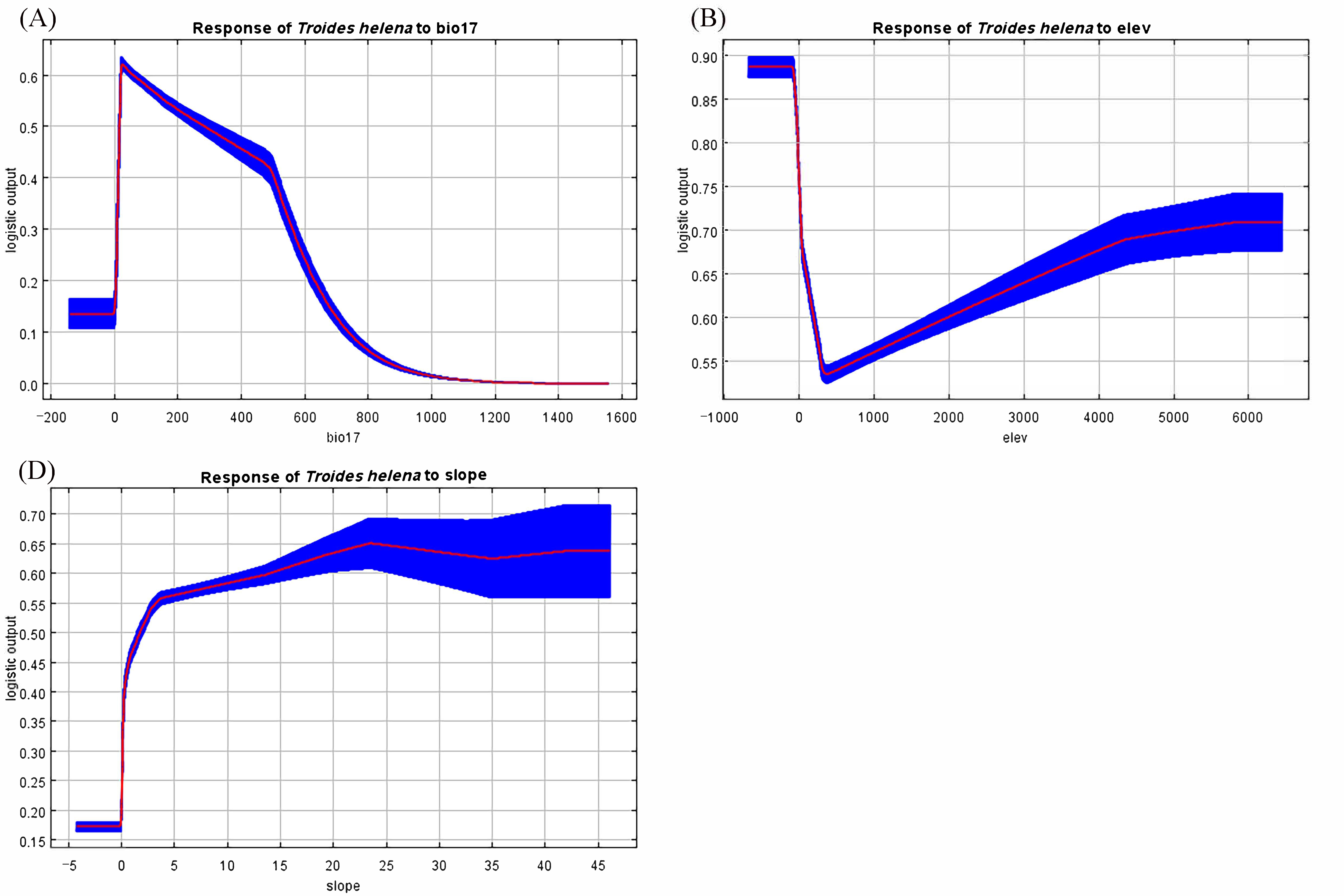

Supplement: Supplementary file 1 [file insects-16-00079-s001.zip › Figure S1.tif]
